# Modeling Parkinson’s disease in LRRK2 rodents

**DOI:** 10.1042/NS20220040

**Published:** 2023-08-16

**Authors:** Chiara Domenicale, Stefano Magnabosco, Michele Morari

**Affiliations:** Department of Neuroscience and Rehabilitation, University of Ferrara, 44121 Ferrara, Italy

**Keywords:** dopamine, leucine rich repeat kinase, neurodegeneration, Parkinsons disease, transgenic mice

## Abstract

Mutations in the leucine-rich repeat kinase 2 (*LRRK2*) gene are associated with familial and sporadic forms of Parkinson’s disease (PD). Sporadic PD and LRRK2 PD share main clinical and neuropathological features, namely hypokinesia, degeneration of nigro-striatal dopamine neurons and α-synuclein aggregates in the form of Lewy bodies. Animals harboring the most common LRRK2 mutations, i.e. p.G2019S and p.R1441C/G, have been generated to replicate the parkinsonian phenotype and investigate the underlying pathogenic mechanisms. Disappointingly, however, LRRK2 rodents did not consistently phenocopy hypokinesia and nigro-striatal degeneration, or showed Lewy body-like aggregates. Instead, LRRK2 rodents manifested non-motor signs and dysregulated transmission at dopaminergic and non-dopaminergic synapses that are reminiscent of behavioral and functional network changes observed in the prodromal phase of the disease. LRRK2 rodents also manifested greater susceptibility to different parkinsonian toxins or stressors when subjected to dual-hit or multiple-hit protocols, confirming LRRK2 mutations as genetic risk factors. In conclusion, LRRK2 rodents represent a unique tool to identify the molecular mechanisms through which LRRK2 modulates the course and clinical presentations of PD and to study the interplay between genetic, intrinsic and environmental protective/risk factors in PD pathogenesis.

## Introduction

Parkinson’s disease (PD) is a progressive, multifactorial, multisystem and clinically heterogeneous motor disorder presenting with cardinal hypokinetic motor symptoms (typically akinesia/bradykinesia, rigidity and tremor) accompanied by a range of neuropsychiatric, cognitive, sensory and autonomic disturbances [1,2]. Main neuropathological features are the loss of nigro-striatal dopamine (DA) neurons and the appearance of proteinaceous aggregates named Lewy bodies (LBs) mainly composed of phosphorylated α-synuclein (α-syn) in dopamine (DA) and non-DA neurons. The disease progresses through three main phases: an early, asymptomatic preclinical phase where neurodegeneration starts but is functionally compensated, followed by a prodromal/premotor phase, where non-motor symptoms such as REM behavior sleep disorders (RBD), depression and hyposmia emerge, and a motor phase where classical hypokinetic symptoms appear and clinical diagnosis is made [1,3]. The etiology of PD is multifactorial and relies on the interplay between intrinsic variables (e.g. age and sex) with genetic and environmental factors. Although >90% of PD cases are classified as sporadic, genome-wide association studies (GWAS) have revealed that the contribution of genetic factors might slightly exceed one-third of PD cases [4]. Indeed, in addition to causative genes associated with typical monogenic forms, about 90 risk loci have been identified that build up an individual polygenic risk affecting age at onset [5], disease course [6] and penetrance [7].

Mutations in the leucine-rich repeat kinase 2 (*LRRK2*) gene are the most common genetic cause of familial PD (4%) and are present in 1% of sporadic PD cases [8–10]. LRRK2 is a multidomain protein with a central catalytic core encompassing a kinase and a GTPase domain surrounded by protein–protein interaction domains. Pathogenic mutations occur in the kinase (p.G2019S, p.I2020T) and GTPase (p.R1441C/G/H) enzymatic domains, resulting in an increase of kinase activity that appears to be instrumental to the neurotoxic effects of the mutant protein *in vitro* [11,12] and *in vivo* [13–16]. The prevalence of LRRK2 pathogenic mutations in the PD population varies depending on country and ethnicity. A recent review covering 291 studies from 51 countries [17] confirms that the p.G2019S mutation has the highest prevalence, with estimates of >30% in Northern Africa (Algeria, Libya, Morocco and Tunisia). Conversely, the p.R1441C/G prevalence is close to 1% with two peaks in Belgian (2% p.R1441C) and Spanish (5% p.R1441G) populations. p.R1441H has <1% prevalence in all countries whereas the p.I2020T variant is much rarer (0.037%). GWAS studies have pointed out that LRRK2 itself represents a risk factor for idiopathic PD [18].

Several models have been generated to investigate the contribution of LRRK2 to PD pathogenesis, spanning from nematodes (*Caenorhabditis elegans*) and flies (*Drosophila melanogaster*) to rats and mice [19–22]. Models with clear construct validity are those carrying PD-associated pathogenic LRRK2 mutations. LRRK2 knock-out (KO) and kinase-dead (KD) rodents do not have construct validity but are useful to interrogate endogenous LRRK2 as a risk factor in sporadic PD and LRRK2 kinase activity as the culprit in neurotoxicity. Moreover, based on the findings that endogenous wild-type (WT) LRRK2 is activated in sporadic PD [23] and a risk variant for sporadic PD in non-coding regions of LRRK2 locus (rs76904798^T^) [18] is associated with increased LRRK2 expression in a specific cell population (monocyte-derived microglia-like cells) [24,25], LRRK2 WT overexpressors (OE) might provide useful information on the role of LRRK2 in sporadic PD. The present review aims to cover the studies performed in rodents carrying PD-associated pathogenic LRRK2 mutations, grouping them into four main sections: PD neuropathology, motor signs, non-motor signs and neurotransmission.

## PD neuropathology

PD is neuropathologically characterized by a progressive neurodegeneration affecting DA and non-DA neurons, and the appearance of α-syn aggregates in the form of LBs and Lewy neurites in dying/surviving neurons. Moreover, phosphorylated tau (ptau) has been shown to co-aggregate with α-syn in a substantial proportion of LBs of the PD brain [26,27], suggesting that tau might play a role in PD or, at least, in some PD subtypes. In fact, tau has been shown to facilitate α-syn aggregation [28]. Finally, evidence of microglial activation in PD patients was collected from post-mortem brains, serum, CSF markers and *in vivo* imaging [29–31], indicating that neuronal loss is associated with a neuroinflammatory response. Some imaging studies also suggest that neuroinflammation occurs early in PD and might even precede neuronal cell loss [32,33].

### Degeneration of nigro-striatal DA neurons

#### Transgenic rodents

Overexpression of human G2019S (hG2019S), R1441C/G (hR1441C/G) or I2020T (hI2020T) has been achieved through bacterial artificial chromosome (BAC) or heterologous promoters. Models are therefore quite heterogeneous in terms of levels, pattern and timing (i.e. conditional vs constitutive) of transgene (TG) expression. Nonetheless, most transgenic rodents do not show overt nigro-striatal neurodegeneration. In detail, stereological analysis showed no loss of DA cells up to 24 months in the Substantia Nigra compacta (SNc) of BAC mice overexpressing hG2019S [34–36], mouse G2019S (mG2019S) [37] or hR1441G [35,38,39] (Table 1). TG levels were reported to be 2.5- to 14-fold higher than endogenous LRRK2 levels. Lack of nigro-striatal neurodegeneration was also observed in hG2019S [40–43] and hR1441C [42] rats, where TG expression levels ranged 2 to 12-fold above endogenous LRRK2 levels (Table 2).

**Table 1:** Studies in LRRK2 BAC transgenic mice; ↓ = decrease, ↑ = increase n.c. = no change

	Mutant/Expression system	Fold TG levels	SNc DA neuron loss/age	DA transmission abnormalities	Phenotype	Pathology
**Li, 2009** [38]	**hR1441G**	5–10	NO 6–12 m	↓ Size/dendrites SNc DA neurons ↓ Nomifensine-induced DA release	↓ Rearing @12 m (DOPA rescue)	↑ ptau IR brain
	**hWT**	5	–	–	Hyperkinetic @10 m	–
**Li, 2010** [37]	**mG2019S**	6	NO 6–12 m	↓ DA content ↓ DA release and ↓ *V*max DA uptake, ↓ repetitive DA release (FSCV in 12 m slices)	NO	n.c. α-syn, UB IR @18 m
	**mWT**	6	–	n.c. DA content, ↑ DA release (FSCV in 12 m slices)	Hyperkinetic @6, 12 m	↓ptau
**Melrose, 2010** [34]	**hG2019S**	2.5–14	NO 22–24 m	n.c. basal and raclopride-induced DA content @18 m ↓ basal DA release (microdialysis) @8–10 m n.c. amphetamine response n.c. DAT IR, D1R, D2R density @18 m	↑ Speed ↑ Thigmotaxis @7–8 m	n.c. α-syn and pS129 α-syn ↑ ptau @18–24 m
	**hWT**	3.5	NO 22–24 m	↓ DA release (microdialysis), n.c. amphetamine response n.c. DAT IR ↑ D1R density	Normal @7–8 m	↑ ptau in hippocampus
**Bichler, 2013** [44]	**hR1441G** see [38]	–	–	–	↓ Rearing @20 m Activity: ↑ @12 m, ↓ @20 m n.c. cognition (passive avoidance) n.c. anxiety (EPM) n.c. depression (FST, TST) n.c. olfaction	–
**Dranka, 2013** [45]	**hR1441G** see [38]	–	–	–	↓ Pole, rotarod @16m n.c. locomotion, gait	–
**Dranka, 2014** [39]	**hR1441G** see [38]	–	–	–	n.c. pain threshold Hyposmia @15m	–
**Volta, 2015** [46]	**hG2019S** See [34]	–	–	–	↑ Activity and rearing @<6 m, n.c. @12 m ↓ NOR @6–12 m	–
	**hWT** See [34]	–	–	–	↓ Locomotion, rearing and NOR @12 m	–
**Kozina, 2018** [35]	**hR1441G** see [38]	–	NO 24 m	–	–	–
	**hWT** see [38]	–	NO 24 m	–	–	–
**Pischedda, 2021** [47]	**hG2019S** See [34]	–	–	–	↓Pole, beam, rotarod test n.c. locomotion @6–12 m ↓ NOR @6–18 m Threalose reversal	n.c. α-syn IR @6m
**Skiteva, 2022** [36]	**hG2019S**	–	NO 10–12 m	n.c. firing DA neurons	↑ Activity and rearing @10–12 m n.c. beam, pole test n.c. EPM, Light/Dark test	–

**Table 2:** Studies in LRRK2 BAC transgenic rats; ↓ decrease, ↑ increase, n.c. = no change

	Mutant/Expression system	Fold TG levels	SNc DA neuron loss/age	DA transmission abnormalities	Phenotype	Neuropathology
**Zhou, 2011** [43]	**hG2019S tetO**	–	NO	n.c. basal DA release and ↓ Response to amphetamine/nomifensine	↑ Locomotion ↓ Response to amphetamine/nomifensine @18 m	–
**Walker, 2014** [40]	**hG2019S**	2	NO	n.c. DAT, TH, VMAT2 PET study	↓ Rotarod @6 m n.c. beam, drag test	TSPO PET no microgliosis see [48]
**Lee, 2015** [41]	**hG2019S**	5–8	NO 12 m	n.c. 5-HT, DA content @12 m Dysmorphic SNc DA neurons @12 m	Postural instability @8 m ↑ Rearing @12 m	n.c. iba-1, GFAP ↑ iNOS and oxidation markers in DA neurons
**Sloan, 2016** [42]	**hG2019S**	12	NO	↓ DA release (FSCV in 18–22 m slices)	Rotarod test ↑@3–6 m, ↓@18–21 m Cognitive impairment @18–22 m (DOPA rescue)	n.c. α-syn, ptau IR in SNc @18–21 m
	**hR1441C**	4–5	NO	↓ DA release (FSCV in 18–22 m slices) ↓ burst firing DA neurons	↓ Rotarod test @18–21 m Cognitive impairment @18–22 m (DOPA rescue)	
	**hWT**	4	–	↓ DA release n.c. firing pattern DA neurons	Normal @18–21 m	–

Regarding the models where TG overexpression was driven by heterologous promoters (Table 3), lack of nigro-striatal degeneration was observed in mice overexpressing hG2019S under the thymus cell antigen 1 (Thy1) promoter [49], the paired-like homeodomain transcription factor 3/pituitary homeobox 3 (Pitx3) promoter [50] or the cytomegalovirus (CMV) enhanced (CMVE)/platelet-derived growth factor (PDGF) promoter [35]. These mice showed similar TG levels (6- to 8-fold) but different patterns of TG expression, ranging from no expression [49] or selective expression [50] in SNc. Lack of nigral DA cell loss was also observed in mice overexpressing hR1441C under the ROSA26 promoter (1.3-fold conditional expression in SNc) [51] or hI2020T under the CMV promoter (1.3-fold expression) [52]. Only a handful of hG2019S LRRK2 transgenic mice showed a late onset (12–24 months) nigral neurodegeneration (18–50%) [53–55]. These mice carried either a neuron-specific hybrid CMVE/PDGF promoter leading to 3-fold TG expression in SNc neurons [53,54] or a tyrosine hydroxylase (TH) promoter driving a conditional 3- to 6-fold TG expression (TetO system) selectively in dopaminergic areas [55]. With the exception of [53], nigro-striatal neurodegeneration was confirmed by the loss of striatal TH-positive terminals as assessed by the analysis of striatal optical density [54], DA content or DA terminal markers [55] and also in vivo imaging [54,56]. When the CMVE/PDGF promoter was used to overexpress hR1441C, either no loss [53] or mild reduction (30%) [56] of nigro-striatal DA neurons was observed.

**Table 3:** Studies in LRRK2 transgenic mice. ↓ = decrease, ↑ = increase, n.c. = no change, GS = hG2019S, STR = striatum, Cx = cerebral cortex

	Mutant/Expression system	Fold TG levels	SNc DA neuron loss/age	DA transmission abnormalities	Phenotype	Pathology
**Lin, 2009** [57]	**hG2019S** CaMKII TetO	8–16 No SNc	–	–	↑ Locomotion n.c. rotarod test @12 m	n.c. Iba-1, GFAP @20 m LRRK2 GS, WT, KD ↑ A53T-induced STR/Cx Degeneration and αsyn pathology GS ↑A53T-induced microgliosis
**Ramonet, 2011** [53]	**hG2019S** CMVE/PDGF	3	18% 19–20 m	n.c. DA content, ↑ 5-HT content in PFCx @15 m	n.c. locomotion	n.c. α-syn and pS129 α-syn, tau @24 m ↑ Autophagic vacuoles and aggregated mitochondria @17 m
	**hR1441C** CMVE/PDGF	3–5 No SNc	NO 20–21 m	n.c. DA content @19–20 m ↓ DA, NE Cx @17 m	↓ Locomotion @15 m	↑ Autophagic vacuoles @17 m
**Daher, 2012** [58]	**hG2019S** CMVE-PDGF see [53]	–	NO 13–14 m	–	–	GS does not enhance A53T αsyn -induced pathology and astrogliosis
**Chen, 2012** [54]	**hG2019S** CMVE/PDGF	High	30–50% 12–16 m	↓ TH density ↓ DAT (SPECT) @16 m	↓ Locomotion @12–16 m (DOPA rescue)	n.c. αsyn, ↑ ptau in SN only No extranigral degeneration
**Herzig, 2012** [49]	**hG2019S** Thy1	High no SNc	NO 19 m	–	↑ Rotarod test @4 m but not 10 m	n.c. αsyn and pS129 αsyn, iba1, tau @15 m GS does not accelerate A53T pathology and astrogliosis
**Maekawa, 2012** [52]	**hI2020T** CMV	1.3	NO 18 m	↓ DA content @3 m	Transient motor deficit @6–10 m	↑ Microtubule polimerization n.c. pS129 α-syn, ptau
**Chou, 2014** [59]	**hG2019S** CMVE/PDGF see [54]		NO 8–9 m	↓ SNc DA firing ↓ DA release @8 m (FSCV)	↓ Locomotion @8–9 m (DOPA rescue)	–
**Tsika, 2014** [51]	**hR1441C** ROSA26 DAT/Cre	1.3 selective expression in SNc	NO	n.c. DA, 5HT content @12 m	n.c. @10–20 m	n.c. α-syn, pS129 α-syn, tau, iba-1/GFAP p62, LC3 @ 12–22 m
**Liu, 2015** [50]	**hG2019S** Pitx3 TetO see [57]	6 selective expression in SNc	NO	↓ STR DA terminals ↓ DA content @12 m ↓ DA release & n.c. DA uptake (FSCV slice) DAT, VMAT2, TH levels: ↑@1 m ↓@18 m	n.c. up to 18 m	n.c. iba1, GFAP IR @20 m
**Weng, 2016** [56]	**hR1441C** CMVE/PDGF see [54]	2	30% 18 m	↓ DA terminals (PET) ↓ DA release (FSCV)	↓ Locomotion, rearing @16–20 m (DOPA rescue)	No extranigral degeneration n.c. asyn, pS129 α-syn, ptau @16 m
**Xiong, 2017** [22]	**hG2019S** CaMKII tetO	5–17 Forebrain 4 Midbrain	NO 22 m	n.c. DA neuron Size and morphology n.c. STR DA terminals @22 m	n.c. locomotion, pole and rotarod test @22 m ↓ Response to amphetamine	↑ HMW α-syn and pS129 α-syn (age-dependent)
**Xiong, 2018** [55]	**hG2019S** TH tetO	3–6 Selective in DA/NA neurons	20–40% 15–24 m	↓ DA terminals & DA content @24 m	↓ Pole test @24 m (DOPA rescue) ↓ Response to amphetamine n.c. rotarod test, locomotion @24 m	↓ NE neurons in LC @24 m ↑GFAP in SN/STR but not Cx @24 m n.c. CD68 IR ↑pS129 α-syn IR @24 m ↑pS129 α-syn WB @15–24 m
**Kozina, 2018** [35]	**hG2019S** CMVE/PDGF see [53]	–	NO 24 m			No microgliosis
**Kim, 2021** [152]	**hG2019S** CaMKII tetO see [22]	–	–	n.c. SNc DA firing ↑ Ca^2+^ currents	–	↑ Translation
**Lim, 2018** [60]	**hG2019S** CMVE/PDGF see [53]	–	–	↓ TH levels @16–21 m	↓ Rotarod test @16–21 m ↑ Anxiety from 11 m ↑ Depression from 2 to 5 m	–
**Arbez, 2020** [61]	**hG2019S** PrP	5	–	–	↓ Rearing and locomotion ↓ Rotarod test @6–12 m	–

#### KI mice

As discussed above, transgenic models are heterogeneous in terms of TGs and promoters used, hence levels and patterns of LRRK2 mutant overexpression. Another confounder is the expression of endogenous LRRK2 in the background. Finally, most studies in BAC rodents lack TG controls, i.e. rodents that overexpress human or murine WT LRRK2. Knock-In (KI) mice are devoid of such confounders and better replicate the genetic architecture of PD patients where mutant LRRK2 is expressed at physiological levels. Both G2019S [62–64] and R1441C/G [65–68] KI mice failed in phenocopying nigro-striatal DA loss, even up to 24 months of age.

### Degeneration of non-DA neurons

Whether and to what extent pathogenic LRRK2 mutations affect non-dopaminergic systems has been poorly investigated. No overt lesion in the cerebral cortex, striatum and cerebellum was revealed by Jade C/Caspase 3 or NeuN immunoreactivity (IR) in 20-month-old CaMKII hG2019S mice [57], 16-month-old CMVE/PDGF hG2019S mice [54] or hR1441C mice [56], even in the presence of 30–50% nigral DA neuron loss [54,56] (Table 3). Conversely, careful stereological analysis found a reduction of the number of noradrenaline (NA) neurons in the Locus Coeruleus of 24-month-old hG2019S/TH conditional mice, in parallel with a 20–40% nigral DA neuron loss [55] (Table 3).

### α-Syn and tau pathology

#### Transgenic rodents

α-syn and phosphoSerine129 (pSer129) α-syn levels measured by immunohistochemical technique were found to be unchanged in BAC mG2019S or hG2019S mice [34,37,47], BAC hR1441C mice [51,56], or BAC hG2019S and hR1441C rats [42]. A similar finding was reported in CMVE/PDGF hG2019S [53,54], Thy1 hG2019S [49] or CMV/hI2020T [52] mice. Only one model, i.e. mice conditionally overexpressing hG2019S under the TH promoter [55], showed an increase of pSer129 α-syn striatal IR and immunoblot levels of pSer129 α-syn, and high molecular weight (HMW) α-syn species in the striatum and ventral midbrain at 15-24 months. Increased ptau IR was occasionally reported in the brain of 20-month-old BAC hG2019S mice [34], in the striatum and cortex of BAC hR1441G mice [38] and, selectively, in the SNc of CMVE/PDGF hG2019S mice [54]. Nonetheless, the majority of studies in transgenic rodents revealed no change in tau or ptau IR [42,49,51–53,56].

#### KI mice

Different studies evaluated α-syn and/or pSer129 α-syn IR in KI mice (Table 4). No change was reported in the midbrain or other brain regions of G2019S KI mice up to 20 months [62,63]. An increase of pSer129 α-syn levels was observed in striatal homogenates of 12-month-old G2019S KI mice which, however, was not confirmed using DAB immunohistochemistry in striatal slices [69]. Most R1441C/G KI mice did not show changes of α-syn levels up to 28 months [65–67]. Remarkably, however, one study reported higher levels of α-syn oligomers in the striatum and cortex of 18-month-old R1441G KI mice compared with WT controls, using immunohistochemistry and immunoblot analysis [68]. Higher oligomer deposition was associated with defective α-syn clearance via chaperone-mediated autophagy [68]. Most studies failed to demonstrate an elevation of tau or ptau IR in KI mice [62,65–67]. Only one study reported an increase of ptau IR in some brain regions, including striatum and midbrain, of old G2019S KI mice which was confirmed by immunoblot analysis of brain lysates [63].

**Table 4:** Studies in LRRK2 Knock-in (KI) mice. ↓ = decrease, ↑ = increase, n.c. = no change

	Mutant	SNc DA neuron loss/age	DA transmission abnormalities	Phenotype	Pathology
**Tong, 2009** [65]	**R1441C**	NO 22 m	n.c. TH, DA content @12–23 m n.c. firing DA neurons but ↓ Response to D2 agonists ↓ Catecholamine release (chromaffin cells)	NO ↓ Response to amphetamine and quinpirole	n.c. α-syn, pS129 α-syn IR and levels @24 m n.c. tau, GFAP IR
**Herzig, 2011** [62]	**G2019S**	–	n.c. DA content n.c. TH, DAT, DARPP32 IR @22 m	NO n.c. response to cocaine @5 m	n.c. α-syn, tau, GFAP, iba1 IR brain @20–22 m
**Yue, 2015** [63]	**G2019S**	NO 18–20 m	↓ DA content @18 m ↓60% basal and amphetamine-evoked DA release (also HET) @12 m	↑ Performance OF, rotarod test @6 m but not 12 m	n.c. α-syn, iba1 IR @>18 m ↑ ptau IR and levels Dystrophic mitochondria @15 m
**Liu, 2014** [66]	**R1441G**	NO 18–22 m	n.c. [^3^H]-DA uptake but ↑ response to reserpine n.c. DAT, VMAT2 levels (WB)	NO 18 m	n.c. mitochondrial markers
**Longo, 2017** [64]	**G2019S** see [62]	NO 19 m	n.c. DA release ↑ *V*_m__ax_ [^3^H]-DA uptake and DAT levels (WB) @12 m ↑ *V*_max_ [^3^H]-DA uptake and ↓VMAT2 levels (WB) @12 m	NO 12 m	–
**Giesert, 2017** [67]	**R1441C**	NO >24 m	–	↓ Pole, beam, gait Hyposmia @ 24 m n.c. anxiety	n.c. ptau hindbrain n.c. αsyn IR midbrain
**Ho, 2020** [68]	**R1441G** see [66]	–	–	–	↑ αsyn oligomers @18 m
**Volta, 2017** [70]	**G2019S** see [63]	–	↑ DA release @<6 m but not >12 m (FSCV in slices) ↑ DAT levels (WB) @>12 m	↑ Rearing @<6 m	–
**Crown, 2020** [71]	**G2019S**	–	–	n.c. rotarod test @8–9 m EcoG and sleep disturbances	–
**Domenicale, 2022** [69]	**G2019S** see [62]	–	↑ *V*_max_ [^3^H]-DA uptake and DAT levels @ 9–18 m ↓VMAT2 levels (WB) @12 m	–	↑ pS129 α-syn levels (WB) @12 m n.c. pS129 α-syn IR
**Hussein, 2022** [72]	**G2019S** see [73]	–	–	↓ Executive functions (5CSRT task) @2–3 m (Donepezil rescue)	–
**Xenias, 2022** [74]	**G2019S** see [63]	–	↓ Striatal DA release (FSCV) @3–4 m	n.c. rotarod test @2 m	
	**R1441C** see [65]	–	↓ Striatal DA release (FSCV) @3–4 m	n.c. rotarod test @2 m but ↓ learning after eticlopride ↑ PKA	

### Neuroinflammation

Several studies have monitored neuroinflammation in the brain of LRRK2 rodents, using Iba1 and CD68 as markers of microglia and glial fibrillary acidic protein (GFAP) of astrocytes. No microgliosis was reported in BAC hG2019S mice [75] or rats [41] (Tables 1 and 2), transgenic mice [49–51,55,57] (Table 3) or KI mice [15,62,63] (Table 4). Consistently, two PET in vivo/ex vivo studies in BAC hG2019S rats [48] and G2019S KI mice [15] showed no genotype differences in the uptake of [^11^C]-PBR28 and [^18^F]-VC701, two ligands of the outer mitochondrial membrane 18-kDa translocator protein (TSPO) which is up-regulated in microglial and astrocytes during the proinflammatory state. BAC hG2019S mice [75] and rats [41] (Tables 1 and 2), transgenic mice [49,50,57] (Table 3) and KI mice (Table 4) [15,62,65] also showed no astrogliosis. In only one study [55], an increase in GFAP IR, confirmed by immunoblot analysis, was found in the striatum and SN of 24-month-old TH hG2019S conditional mice.

### Do LRRK2 rodents recapitulate PD neuropathology?

The studies detailed above clearly show that BAC rodents and KI mice do not develop nigro-striatal DA degeneration over their life course. Among transgenic mice carrying heterologous promoters, instead, only those expressing hG2019S (or hR1441C) under the CMVE/PDGF [53,54,56] or the TH [55] promoters showed late onset neurodegeneration [53–56]. Age at examination, expression strategy, TG patterns and levels in the brain and SNc cannot explain such discrepancy. A common denominator that would explain the success of these two models in phenocopying the nigro-striatal degeneration is also not evident. In fact, the two models differ for promoter, expression strategy (constitutive vs. conditional) and patterns of TG expression (diffuse throughout the brain vs. selective in catecholaminergic areas). We should also consider that not all CMVE/PDGF-driven hG2019S and hR1441C TG mice showed neurodegeneration [53,58]. Therefore, the possibility that a hitherto unrecognized subtle genetic or environmental modulatory factor contributes to accelerate DA neuron loss in these neurodegeneration-prone LRRK2 models should be considered. Indeed, studies in LRRK2 models suggest that LRRK2 mutation alone is not sufficient to cause DA neuron loss, questioning the face validity of LRRK2 rodents as models of the clinical phase of PD. Consistent with this view, typical α-syn aggregates have never been observed in LRRK2 transgenic rodents or KI mice, including those showing neurodegeneration [53–56]. However, since the LRRK2 mutations have low penetrance and LBs are not always detectable in the brain of LRRK2 PD patients [76], one could argue that the processes of α-syn aggregation and neurodegeneration would be much delayed in LRRK2 rodents, never appearing during the life course. Indeed, clues that α-syn turnover is altered in LRRK2 rodents might be provided by the two studies showing elevated levels of HMW α-syn species in aged R1441C KI [68] and TH hG2019S [55] mice. Again, however, considering the striking differences between these two models, and that most studies in LRRK2 rodents failed to detect changes of α-syn turnover, it remains difficult to identify the variables that would make these mice a reliable model of (initial) α-syn pathology. Lack of microgliosis confirms that LRRK2 mice do not model the overt phase of PD since samples from sporadic PD patients show evidence of microglial activation [29–31] and both sporadic PD and, to a lower degree, LRRK2 PD patients present with a proinflammatory blood markers profile [77,78]. In conclusion, no LRRK2 rodent fully replicates the clinical phase of PD. However, taking into account that TH hG2019S conditional mice show degeneration of SNc DA and LC NA neurons, elevation of HMW α-syn along with astrocytosis, these mice represent the current LRRK2 model that most faithfully replicates PD neuropathology. Further studies are awaited to confirm the face validity of this model.

## Motor signs

Cardinal features of PD are bradykinesia, rest tremor and/or rigidity [1,2]. These symptoms are present in the early clinical phase of PD and are predominantly due to functionally uncompensated degeneration of nigro-striatal DA neurons. In fact, these symptoms respond to dopaminergic therapy. Instead, postural instability, falls, freezing of gait, dysphagia, and other motor deficits are typical of the more advanced stages of the disease [1,2]. The motor phenotype of LRRK2 rodents has been widely investigated with different tests in different contexts. Spontaneous activity was measured in an open field (OF) or smaller environment (e.g. cylinder test and home cage). Stimulated activity was analyzed using the rotarod test or more sensitive tests for coordinated activity (e.g. pole test, balanced beam and drag test). Gait parameters were analysed using the footprint test whereas muscle strength using the inverse grid.

### Transgenic rodents

No difference in motor performances between 7- and 8-month-old BAC hG2019S mice and hWT LRRK2 OE or non-transgenic controls were originally reported in the balanced beam, inverse grid, and footprint tests [34] (Table 1). Subtle differences emerged only in the OF test where transgenic mice walked faster and tended to explore closer to the walls than controls (increased thigmotaxis) [34]. This was recently replicated in 10- to 12-month-old BAC hG2019S mice, that showed an increase in exploratory activity but no deficits in motor balance or coordination compared with non-transgenic littermates [36]. Consistently, an increase of rearing and horizontal activity was found in 4- to 6-month-old BAC hG2019S mice compared with non-transgenic controls [46]. In line with a previous study [79], BAC hWT LRRK2 OE were hypokinetic but while the hG2019S-associated hyperkinesia vanished at 12 months [46] the hWT-associated hypokinesia was long-lasting [79]. In contrast with these studies, an age-dependent motor impairment was recently described in BAC hG2019S mice [47]. Although these mice had similar exploratory behavior as non-transgenic controls, they showed a reduction of motor activity and coordination in the pole test, rotarod test and balanced beam test starting from 6 up to 18 months [47]. In the only study dealing with BAC mG2019S mice, no difference in motor activity was observed between non-transgenic controls while mWT OE were hyperkinetic [37]. Studies in BAC hR1441G mice led to more consistent results, showing a reduction of rearings at 12 months [38] or 20 months [44], and an impairment of pole and rotarod performance at 16 months [39]. Spontaneous activity and gait parameters were unaltered [39] or showed a biphasic course with an increase at 12 months and a reduction from 16 months onwards [44]. More relevant, motor deficits were rescued by L-DOPA or apomorphine pointing to an underlying deficit in dopaminergic transmission [38].

Initial study in BAC hG2019S rats (Table 2) revealed that conditional overexpression of hG2019S from 5 months of age caused late hyperactivity at 18 months whereas constitutive overexpression of hG2019S did not produce behavioral changes [43]. Later studies showed that constitutive hG2019S overexpression was associated with a reduction of rotarod activity, but not performance in the beam and drag tests, at 6 months [40]. Interestingly, in a study comparing the impact of different LRRK2 mutations, a biphasic pattern of motor activity, i.e. increase at 3–6 months and decrease at 18–21 months, was reported in BAC hG2019S rats whereas only a reduction at 18–21 months was observed in BAC hR1441C rats [42]. Finally, increased rearing activity was found in 12-month-old BAC hG2019S mice compared with non-transgenic controls [41].

Conditional mice where hG2019S overexpression was controlled by a TetO system under the calcium-calmodulin-dependent protein kinase II (CaMKII) promoter showed elevated locomotion at 12 months [57] (Table 3). Enhanced rotarod performance was observed in 4-month-old but not 10-month-old Thy1 hG2019S mice [49]. Finally, mice expressing hG2019S under the mouse prion protein promoter (PrP), that drives the TG expression in all neurons (but Purkinje cerebellar cells) and glia [80], showed a reduction of rearing, locomotion (OF) and rotarod performance at 6-12 months [61]. Conversely, Pitx3 hG2019S mice [50] and ROSA26 hR1441C mice [51] (that selectively express the TG in midbrain dopaminergic areas) were not phenotypic. Regarding those models showing nigro-striatal neurodegeneration, original study in CMVE/PDGF hG2019S mice [53] failed to observe motor changes, possibly due to the low degree of nigral DA cell loss (18%). Two more recent studies in the same model, instead, reported late (12–21 months) reduction of spontaneous locomotion [54] or rotarod performance [60]. Also CMVE/PDGF hR1441C mice were less mobile in an OF at 12–20 months, irrespective of nigro-striatal DA loss [53,56]. Reversal by L-DOPA confirmed the dopaminergic nature of these motor deficits [54,56]. Finally, motor impairment in the pole test without gross changes in spontaneous activity was reported in conditional TH hG2019S mice at 24 months [55].

Collectively, studies on motor behavior in LRRK2 transgenic rodents greatly differ in motor tests and contexts, age at examination, TG expressed and genetic background of the animal and, in general, model used. Although at a first glance a feeling of great inconsistency prevails, a more in-depth analysis reveals some general features: (i) reduced motor activity in hR1441C/G OE >12 months [38,42,53,56,44,45]; (ii) biphasic changes of motor activity in hG2019S OE, with elevated performance in <12-month-old rodents [34,36,41,42,49,57,46] and hypokinesia in older ones [42,54,55,47,60].

### KI mice

Studies in G2019S KI mice compared with WT controls (Table 4) revealed no changes in spontaneous locomotion in motility cages at 5 months [62] or rotarod performance at 2 months [74]. On the other hand, G2019S KI mice outperformed WT controls in the OF and rotarod test at 6 months [63,81,70], a finding that, however, was not confirmed by more recent studies [64,71]. R1441C/G KI mice do not show differences in spontaneous locomotion or rotarod performance compared with WT counterparts up to 22 months [65,66,74]. However, subtle motor deficits in the pole and beam tests along with gait abnormalities appear in R1441C KI mice at 24 months [67].

### Do LRRK2 rodents recapitulate PD motor symptoms?

In agreement with late appearance of motor symptoms in sporadic PD and LRRK2 PD, a number of TG OE manifest late onset hypokinesia. Among BAC mice, this is more consistently observed in hR1441C/G than hG2019S rodents whereas among mice where TG expression is driven by heterologous promoters, those carrying CMVE/PDGF show the most consistent hypokinetic phenotype. LRRK2 KI mice have, if any, a mild motor phenotype. Similar to hG2019S OE, young G2019S KI animals show elevated performance [63,81,70] but different from hG2019S OE, aged G2019S KI mice never develop motor deficits. Similar to hR1441C/G OE, one study reported hypokinesia in aged R1441C mice [67] although in other studies, R1441C/G mice do not show a motor phenotype. Motor impairment is associated with nigro-striatal DA loss in some [54–56] but not all [67,44,59] studies, suggesting that hypokinesia might be associated with early dysfunction of the nigro-striatal dopaminergic pathway or early compensatory changes of non-dopaminergic (e.g. glutamatergic) pathways.

## Non-motor signs

In keeping with the view that PD is a multisystem disorder, a wide range of non-motor symptoms complicate the clinical picture of PD patients [82,83]. Non-motor symptoms encompass sensory disturbances (e.g., hyposmia, visual hallucinations, pain), neuropsychiatric symptoms (e.g., anxiety, depression, dementia, apathy and fatigue), sleep disorders (e.g., insomnia, sleep attacks, REM sleep behavior disorders or RBD) and autonomic dysfunctions (e.g. nocturia, constipation, orthostatic hypotension) [82,83]. Hyposmia, RBD, constipation, depression can antedate typical motor symptoms by several years and represent behavioral signatures of the prodromal phase of PD [3,82,83]. The neurobiological base of non-motor symptoms is complex as it is not always possible to find a single cause [84]. Some non-motor symptoms are sustained by the nigro-striatal degeneration and respond to some extent to dopaminergic drugs whereas the majority of them are sustained by the degeneration of acetylcholine (ACh), NA, serotonin (5-HT) neurons and respond to non-dopaminergic drugs [84]. Although not deeply and systematically investigated as the motor signs, different non-motor signs have been evaluated in LRRK2 rodents.

### Olfaction

BAC hR1441G mice subjected to the buried or block tests did not show olfactory deficits at 6 and 14 months [44] (Table 1). In fact, they took the same time as non-transgenic controls in retrieving a treat buried under the bedding, keeping the ability to discriminate between familiar and unfamiliar scents [44]. Conversely, hyposmia was reported in 15-month-old BAC hR1441G mice [39] (Table 1). Hyposmia was later confirmed in 24- to 26-month-old R1441C KI mice exposed to a more elaborated experimental protocol where animals were trained to discriminate scents paired with food reward [67] (Table 4). These animals also showed loss of smell discrimination [67].

### Gastrointestinal function

The water content and dry weight of stools of BAC hR1441G mice and non-transgenic controls were compared over a wide time frame, from 2 to 21 months of age [44] (Table 1). No difference between genotypes was observed although during aging these parameters oscillated to a greater extent in transgenic compared with non-transgenic mice, which was interpreted as a sign of dysfunctional activity in mutant mice [44]. However, no changes in stool frequency or water content and wet weight of stools were found in BAC hR1441C and BAC hG2019S rats up to 21 months [42] (Table 2).

### Depression

No difference in depression-related behaviors were observed using the forced swimming test (FST) and tail suspension test (TST) in BAC hR1441G mice across different ages (6–19 months) [44] (Table 1). Conversely, R1441C KI mice struggled longer in the TST and spent less time floating in the FST than WT mice at the age of 8–9.5 months, suggesting resilience to stress and behavioral despair [67] (Table 4). In G2019S KI mice, no difference in sucrose preference was observed between 10-week-old G2019S KI mice and WT controls [85] (Table 4). However, when exposed to social stress due to repeated contact with an aggressor as in the chronic social defeat stress test, G2019S KI mice did not develop anhedonia-like symptoms as WT animals did [85]. A different picture emerged from the analysis of CMVE/PDGF hG2019S mice where a very early (2–5 months) increase in immobility time, an index of behavioral despair, was observed in the FST and TST [60] (Table 3).

### Sleep disturbances

Careful sleep analysis was carried out in 8–10-month-old G2019S KI mice [71] (Table 4). These mice slept a similar amount of time as WT counterparts, but the number of sleep bouts was greater and their duration shorter, indicating sleep fragmentation. Moreover, an increase in spindle density and duration was observed in G2019S KI mice. These events, however, were not reversed by in-diet administration of MLi-2 at an equivalent dose of 60 mg/kg, suggesting LRRK2 kinase hyperactivity was not involved [71]. An increase of time spent in REM sleep was observed in BAC hG2019S mice compared with non-transgenic controls at the age of 12 and 18 months, which was associated with an increase in the levels of the circadian locomotor output cycles protein kaput (CLOCK) protein in the midbrain [86]. Electroencephalography (EEG) patterns and phase transition shifts remained unchanged in these mice. However, following sleep deprivation, LRRK2 mutants showed sleep fragmentation [86].

### Anxiety

Clues on anxiety behavior were obtained from the analysis of exploratory parameters in an OF or from more specific tests such as the elevated plus maze (EPM), light/dark box, and sucrose preference tests. A mild elevation in thigmotaxis, an index of anxiety-like behavior, was originally observed in 7- to 8-month-old BAC hG2019S mice exposed to a novel environment [34]. More recently, however, a reduction of thigmotaxis was reported in 10- to 12-month-old BAC hG2019S mice compared with non-transgenic controls [36]. Nonetheless, the time spent and the number of entries into the light arena (light/dark test) or in the open arms of an elevated maze (EPM test) were similar between genotypes, suggesting that the G2019S mutation was not associated with anxiety behavior [36] (Table 1). BAC hR1441G mice monitored in an OF at different ages (6–19 months) also did not reveal anxiety behavior [44] whereas age-dependent anxiety-like behaviors were observed in CMVE/PDGF hG2019S mice challenged in the EPM, light/dark box and sucrose preference tests [60] (Table 3). Anxiety behavior was age-dependent since it emerged not earlier than 11 months [60]. Indeed, young 3-month-old G2019S KI mice tested in an EPM [85] or R1441C KI mice exposed to an OF [67] did not show anxious phenotype (Table 4).

### Cognitive and executive functions

An age-dependent impairment of long-term recognition memory was revealed in BAC hG2019S mice tested in the novel object recognition (NOR) test [47,46] (Table 1). Indeed, 3-month-old BAC hG2019S mice did not show cognitive deficits when compared with non-transgenic controls whereas 12-month-old BAC hG2019S mice lost the ability to discriminate between a novel and an old object [46]. Memory impairment was confirmed in BAC hG2019S mice using the NOR test in a wide time-window (6–18 months) [47]. Short-term memory was also reduced in the spontaneous alternation test in aged (18–21 months) BAC hG2019S and hR1441C rats compared with non-transgenic and hWT LRRK2 OE [42] (Table 2). Conversely, no learning/memory deficits were observed in 21-month-old BAC hR1441G mice using a passive avoidance protocol [44]. Executive functions were investigated in 2- to 3-month-old G2019S KI mice [72] (Table 4) using the 5-choice serial reaction time task (5CSRTT), a test where the animal is given a food reinforcement upon correct selection of a visual stimulus appearing in one (focused attention) or five (divided attention) windows. This test informs on visuospatial attention and impulsivity. Significant increases in the percentage of omitted response and response latencies were observed in mutant mice in the absence of motivation or motor abnormalities [72], consistent with deficits in divided attention. This deficit was reversed by the acetylcholinesterase (AChE) inhibitor donepezil pointing to an underlying deficit in cholinergic transmission. Under a different reinforcement schedule, G2019S KI mice also showed goal-directed learning deficits [72].

### Pain

Nociceptive pain threshold was unchanged in BAC hR1441G mice exposed to the formalin test at 6, 9 and 21 months [44] (Table 1). Mechanical pain was also unchanged in 15-month-old BAC R1441G mice challenged in Von Frey filament test [39] (Table 1).

## Neurotransmission

Aberrant transmission at dopaminergic and non-dopaminergic pathways in the PD brain has been revealed by different techniques. Local field potentials recording revealed abnormal patterns of neuronal discharge in the basal ganglia of the PD brain, particularly in the glutamatergic neurons of the subthalamic nucleus [87,88]. Transcranial Magnetic Stimulation, instead, unveiled cortical disinhibition in PD patients, likely reflecting an unbalance between GABAergic and glutamatergic pathways [89]. Finally, positron emission tomography (PET) and single photon emission tomography (SPECT) highlighted changes at dopaminergic and non-dopaminergic [90] pathways, also during the prodromal phase [84,91]. These studies confirmed ongoing degeneration of multiple neuronal populations in the PD brain but also suggested that pathway dysfunction antedates neuronal loss.

### Dopaminergic transmission

#### DA content and release

DA content in striatum has been reported to be unchanged [34,51,53,62,65] or reduced [37,50,52,63,60,92] in transgenic rodents (Tables 1-3) and KI mice (Table 4). However, DA content is a rough estimation of DA stores and does not inform on extracellular DA release dynamics. Therefore, *in vivo* microdialysis was employed to monitor basal and drug-stimulated DA release in the striatum of LRRK2 mice. BAC hG2019S OE showed a 33% reduction of basal DA levels compared with non-transgenic controls [34] (Table 1). Nonetheless, hWT LRRK2 OE showed a larger reduction (66%), possibly questioning whether such dysfunction relied on the overexpression of LRRK2 itself or its kinase activity [34]. *In vivo* microdialysis also revealed a 60% reduction of basal striatal DA levels along with an elevation of DA turnover in 12-month-old homozygous and heterozygous G2019S KI mice, which was not associated with motor deficits or nigro-striatal neurodegeneration [63] (Table 4). Another study, however, failed to detect differences in basal extracellular DA levels between 19-month-old G2019S KI mice and WT controls, instead reporting a reduction of DA turnover in mutant mice [64]. This study also showed that the LRRK2 kinase inhibitor Nov-LRRK2-11 did not affect basal extracellular DA levels in striatum [64].

DA release was also analysed in striatal tissues obtained from LRRK2 mice using fast scan cyclic voltammetry (FSCV). FSCV revealed a reduction of stimulus-induced DA release in striatal slices of CMVE-PDGF hR1441C mice at 18 months, concurrent with DA neuron loss [56]. However, reduction of stimulus-evoked DA release in the absence of nigro-striatal degeneration was found in striatal slices from CMVE-PDGF hG2019S mice [59], BAC mG2019S mice [37] and Pitx3 hG2019S mice [50] at 8–12 months (Tables 2 and 3), suggesting that dysfunction of dopaminergic transmission precedes degeneration of nigral DA cell bodies. In an elegant experiment in BAC mG2019S mice [37], repetitive stimulation caused progressive DA release suggesting defects in vesicle recycling (Table 3). Reduction of DA release was also reported in 18- to 22-month-old BAC hG2019S and BAC hR1441C rats [42] (Table 2). However, reduction of DA release was also observed in hWT LRRK2 OE [42]. Very young BAC hR1441G mice (6–8 weeks), instead, did not show differences in the electrically-evoked striatal DA release compared with WT controls [93].

As far as KI mice are concerned (Table 4), an original amperometry study revealed an impairment of catecholamine release from dissociated chromaffin cells of R1441C KI mice [65], which was recently confirmed in striatal slices of 3- to 4-month-old R1441C KI mice [74]. Likewise, constant amperometry revealed a reduction of stimulated DA release in 3-month-old G2019S KI mice compared with WT controls [74,94] or LRRK2 KO and KD mice [94]. In contrast, FSCV revealed that striatal slices of young (<6 months) G2019S KI mice release more DA than WT slices, a difference that disappears at 12 months [70]. Consistently, no difference in K^+^-stimulated [^3^H]-DA release was found in synaptosomes prepared from the striatum of 3- and 12-month-old G2019S KI mice compared with WT controls [64,95] even when repetitive K^+^ pulses were applied [64] (Table 4).

#### DA transporter function

The functions of membrane DA transporter (DAT) and vesicular monoamine transporter 2 (VMAT2) are essential for DA homeostasis. In fact, dysfunctional transporter activity has been related to nigro-striatal degeneration [96–100]. DAT and VMAT2 uptake has been assessed directly or, indirectly, by measuring DA release dynamics in FSCV experiments performed in the presence of DAT or VMAT2 inhibitors. No difference in total [^3^H]-DA uptake was originally reported in synaptosomes from R1441G KI mice compared with WT mice up to 18 months [66] (Table 4). DA uptake kinetics extrapolated from FSCV experiments revealed a reduction of maximal speed (*V*_max_) in 12-month-old BAC mG2019S mice compared with non-transgenic littermates [37] (Table 1) or no change of *V*_max_ in 12-month-old Pitx3 hG2019S mice compared with WT controls [50] (Table 3). A careful [^3^H]-DA uptake analysis in G2019S KI mice, instead, showed an age-dependent 2-fold elevation of *V*_max_ in a striatal synaptosomal preparation from G2019S KI mice compared with WT mice (Table 4). This increase was significant from 9 through 18 months of age and was not paralleled by changes in *K*_m_, indicating no changes in transporter affinity for DA [64,69]. Acute administration of MLi-2 (10 mg/kg, i.p.) could not affect *V*_max_ elevation in G2019S KI mice, possibly suggesting it was not sustained by ongoing LRRK2 activity [69]. Functional DAT changes came along with an increase in striatal DAT protein levels although this was observed in a narrower time frame (9 and 12 months) [64,69,70]. To corroborate the view that DAT changes are time-dependent, the locomotor response to cocaine was unchanged in 5-month-old G2019S KI mice compared with WT controls [62]. Moreover, no difference in DAT IR was found in 18-month-old BAC G2019S transgenic mice compared with non-transgenic controls [34]. Immunoblot technique also revealed biphasic changes of DAT levels in Pitx3 hG2019S mice compared with non-transgenic controls, with elevation at 1 month and decrease at 18 months, although no difference was observed when comparison was made to hWT LRRK2 OE [50].

More consistent, albeit indirect, evidence of dysfunctional DAT activity was obtained from *in vivo* microdialysis studies where striatal DA levels were elevated by DAT blockers such as nomifensine or GBR-12935. In fact, blocking DA reuptake would amplify DA release occurring during spontaneous or stimulus-evoked DA synaptic activity. Nomifensine-induced DA release was impaired in BAC hR1441G mice [38] (Table 1), CMVE/PDGF hG2019S mice [59] (Table 3) and conditional hG2019S rats [43] (Table 2) aged 8–18 months. This was confirmed in 19-month-old G2019S KI mice using GBR-12935 [64] (Table 4). Indirect evidence for dysfunctional DAT might also come from *in vivo* experiments with amphetamine. Amphetamine modulates DA release via complex mechanisms involving DA transporters and trace amine-associated receptors [101,102]. Amphetamine enters nerve terminals via passive diffusion (due to its lipophilicity) and DAT-mediated uptake. Inside the synapse, amphetamine interacts with VMAT2 on synaptic vesicles, triggering DA efflux and/or blocking DA uptake. This leads to the cytoplasmic accumulation of DA, DAT reversal and Ca^2+^-independent DA efflux [102,103]. As DAT substrate, amphetamine also competitively inhibits endogenous DA uptake, elevating DA levels. Amphetamine-stimulated DA release was consistently impaired in TH hG2019S mice [92] (Table 3), conditional hG2019S rats [43] (Table 2), and G2019S KI mice [63] (Table 4). Moreover, taking into account that the locomotor response to amphetamine correlates with the stimulation of striatal DA release, also the blunted locomotor response to amphetamine observed in R1441C KI mice [65] might be an index of blunted DAT activity. In addition to blocking transporters, amphetamine also activates TAAR1, a member of the TAAR family of GPCRs [104,105]. *In vivo* studies have shown that TAAR1 KO mice are hypersensitive to the DA-enhancing action of low doses of amphetamine (2.5–2.9 mg/kg) [106,107] suggesting that TAAR1 activation dampens the catecholamine-releasing action of amphetamine [106,107]. Since this effect might occur via modulation of DAT trafficking [107], it would be interesting to test whether changes in DAT activity in LRRK2 rodents are associated with changes in TAAR1 expression. In contrast with the abovementioned studies, the behavioral and/or neurochemical responses to cocaine and/or amphetamine were found to be unchanged in 5- to 13-month-old G2019S KI mice [62] (Table 4) or even enhanced in BAC hG2019S mice compared with hWT LRRK2 OE and non-transgenic controls [34] (Table 1).

VMAT2 function has been much less investigated than DAT function in LRRK2 rodents. *In vivo* imaging did not reveal changes in the uptake of VMAT2 (and DAT) PET ligands in BAC hG2019S rats, indicating no major changes of target protein(s) [40] (Table 2). Basal [^3^H]-DA uptake in striatal synaptosomes was similar between 3- and 18-month-old R1441G KI mice and WT controls, although bath application of 50 nM reserpine (a VMAT2 blocker) reduced to a greater extent [^3^H]-DA uptake in R1441G KI mice [66] (Table 4). The difference between genotypes, however, was mild and vanished at 10-fold higher reserpine concentrations [66]. [^3^H]-DA uptake was slightly elevated in whole-brain synaptic vesicles of G2019S KI mice compared with WT mice [64] although this was only observed in 12-month-old and not younger or older mice [64,69]. Consistently, however, 12-month-old G2019S KI mice were more resilient to the hypokinetic action of a low dose (1 mg/kg) of reserpine [64]. This would suggest greater intrasynaptic DA levels and/or reduced expression of VMAT2. In fact, VMAT2 levels were ∼50% reduced in 12-month-old G2019S KI mice compared with WT, LRRK2 KO and KD mice [64,69]. VMAT2 expression and levels were also reduced in Pitx3 hG2019S mice at 12 months and 18 months, respectively [50] (Table 3).

#### DA neuron morphology and firing

Morphology of SNc DA neurons was occasionally investigated in LRRK2 rodents. Dysmorphic DA neurons were observed in BAC h1441G mice (reduction of cell body size and number of dendrites) [38] (Table 1) and BAC h2019S rats (elongated shape) [41] (Table 2). Functional studies did not reveal major changes of nigral DA neuron firing in LRRK2 mutants compared with WT controls. In detail, intracellular recording in midbrain slices showed similar firing rates in DA neurons from <2month-old R1441C KI and WT mice [65] (Table 4) although the inhibitory effect operated by D2 agonists in R1441C KI mice was somewhat blunted (see below). Likewise, no difference in the firing rate of SNc (and VTA) DA neurons was revealed by patch-clamp recording in midbrain slices of 10- to 12-month-old BAC hG2019S mice [36] (Table 1) which is in line with the finding that LRRK2 inhibitors IN-1, GNE-7915 and GSK2578215A did not alter DA neuron firing in WT mice [108]. *In vivo* extracellular recording also showed no difference in the firing rate of DA neurons in 16- to 22-month-old BAC hR1441C rats compared with hWT LRRK2 OE and non-transgenic controls [42] (Table 2), although a more regular pattern of activity was observed in mutant OE, possibly due to a reduction in burst firing. This effect was age-dependent as it was not observed at 6 months of age [42].

#### DA receptor activity

No major changes in D1 and D2 receptor density was observed in LRRK2 mice. The density of D1 and D2 receptors investigated by autoradiography was similar between 18-month-old BAC hG2019S mice and non-transgenic controls [34] (Table 1). Conversely, a significant 11% increase in D1 binding was observed in hWT LRRK2 OE, with BAC hG2019S mice only showing a trend to an increase [34]. No change in D1 receptor levels was detected in striatal lysates from 4-month-old G2019S KI mice using immunoblot technique [109] (Table 4). Interestingly, however, after stimulation of receptor trafficking with apomorphine in striatal slices, a reduced amount of D1 receptor IR was noted in the vesicle compartment of G2019S KI mice [109]. This was consistent with a reduced internalization of D1 receptors, a phenomenon also observed in cell lines [109].

D2 transmission was investigated in functional studies. The hypolocomotive response induced by systemic administration of D2 agonist quinpirole was somewhat blunted in R1441C KI mice, indicating D2 autoreceptor down-regulation [65] (Table 4). It is noteworthy that this genotype difference was detected at low but not high doses of D2 agonist indicating a change in sensitivity between genotypes but not an overall impairment of D2 autoreceptor function. Loss of D2 autoreceptor sensitivity was also found in striatal synaptosomes from young BAC hG2019S mice compared with non-transgenic controls [46] (Table 3). In both genotypes, the D2 agonist pramipexole inhibited the K^+^-stimulated [^3^H]-DA release at 0.1 and 1 µM. However, the effect of the lower concentration was much attenuated in BAC hG2019S mice [46]. hWT LRRK2 OE also were found to be unresponsive to a motor inhibiting dose of pramipexole, possibly due to greater D2 autoreceptor saturation/activation [79]. Perhaps in line with this hypothesis, patch-clamp recordings of striatal projection neurons (SPNs) revealed an increase of paired-pulse probability ratio at glutamatergic synapses by remoxipride in 3- to 6-month-old hWT LRRK2 OE compared with non-transgenic controls [79]. In contrast with these studies, but perhaps not too surprisingly considering the experimental readout, DA content and DA turnover after acute raclopride challenge were similar in BAC hG2019S mice and hWT LRRK2 OE [34] (Table 1).

Opposite to D2 autoreceptors, D2 postsynaptic receptors appear supersensitive in G2019S KI mice [110]. Indeed, patch-clamp recording of medium-sized GABAergic SPNs revealed that the D2 agonist quinpirole inhibited both the spontaneous and electrically-evoked extracellular postsynaptic currents (EPSCs) in 6-month-old G2019S KI mice being ineffective in WT mice [110]. This was likely due to LRRK2 kinase hyperactivity since it was not observed in LRRK2 KO and KD mice although it could not be reversed by bath application of the LRRK2 inhibitor GSK2578215A [110]. Mechanistic analysis revealed that postsynaptic D2 receptor activation on striatal G2019S SPNs induces phospholipase C (PLC) activation and endocannabinoid retrograde release leading to a reduction of glutamate (GLU) release from cortico-striatal terminals, hence spontaneous EPSCs [110]. Hypoexcitability of indirect pathway SPNs in 3-month-old R1441C KI but not G2019S KI mice was recently reported using whole-cell current clamp recordings [74]. R1441C mice were also more sensitive to eticlopride than WT controls, since D2 receptor antagonism selectively disrupted motor learning in KI mice, an effect related to cAMP-PKA signaling pathway up-regulation [74].

### Non-dopaminergic transmission

#### Cholinergic system

Deficits in brain cholinergic system were reported in LRRK2 mutant mice. Striatal and cortical cholinergic neurons were found to be significantly less ciliated in R1441C KI mice compared with WT littermates at 7 months [111] which was later confirmed in striatal cholinergic interneurons from 13-month-old G2019S KI mice and 10-month-old BAC hG2019S mice [112]. This might have some implications on PD pathogenesis. In fact, defective cilia are unable to properly signal via the sonic hedgehog (Shh) pathway attenuating glial cell line-derived neurotrophic factor (GDNF) release and neurotrophic support to DA neurons [112]. Morphological abnormalities in brain cholinergic transmission were also revealed in G2019S KI mice [72]. Specifically, reduction of cholinergic fiber density was found in the cerebral cortex and striatum of 2- to 3-month-old G2019S KI mice compared with WT mice which correlated with deficits in executive functions. Interestingly, AChE blockade with systemic donepezil rescued deficits in G2019S KI mice being less effective in WT mice [72].

#### Noradrenergic system

Reduced content of NA, DA and DA metabolites 3,4-dihydroxyphenylacetic acid (DOPAC) and homovanillic acid (HVA) was found in the cerebral cortex of CMVE/PDGF hR1441C mice at 17 months [53] (Table 3).

#### Serotonergic system

Increased 5-HT content was reported in the prefrontal cortex (PFCx) of 14- to 15-month-old CMVE/PDGF hG2019S mice compared with non-transgenic controls [53]. Conversely, no difference in 5-HT content was found in the striatum of ROSA26 hR1441C conditional mice [51], BAC hG2019S rats [41] and CMVE/PDGF hG2019S mice [60] compared with non-transgenic controls, although in CMVE/PDGF hG2019S mice a reduction of hippocampal 5-HT content developed by 12 months. Changes of serotonergic transmission were confirmed by an age-dependent increase in 5-HT1A receptor positive cells along with 5-HT1A levels in dorsal and ventral hippocampus, amygdala and dorsal raphe of middle-age (43–52 weeks) mice possibly reflecting an increased receptor expression [60].

#### Glutamatergic system

Glutamatergic transmission was investigated by measuring GLU levels or analyzing EPSCs in SPNs, that indirectly inform on GLU release from cortico/thalamo-striatal terminals. GLU release from striatal and cortical synaptosomes was similar in 3-month-old G2019S KI and age-matched WT, LRRK2 KO and KD mice [95]. However, the LRRK2 inhibitor IN-1 attenuated the K^+^-stimulated GLU release in the striatum and cortex of WT mice [95,113], an effect replicated using [^3^H]D-Asp as a GLU marker [114]. This suggests a facilitatory control of endogenous LRRK2 on GLU release, possibly via synapsin I phosphorylation [114]. Indeed, an elevation in the frequency of spontaneous EPSC and AMPA-evoked responses was revealed using patch-clamp recording of SPNs in slices of pre-weaning G2019S KI mice, likely reflecting up-regulation of cortico-striatal glutamatergic transmission [73,115]. This increase was LRRK2 kinase-dependent, pathway-independent (i.e. observed both in direct and indirect pathway SPNs) and age-dependent since it was evident at day 21 postnatal but disappeared at 2 months [73]. An age-dependent increase of miniature EPSCs was also confirmed in SPNs of G2019S KI mice by [70], although in a wider time-frame (1–3 months) possibly due to the different mouse genetic background (homozygous vs heterozygous G2019S KI mice) and sex (male and female mixed population vs male mice). In contrast with this finding, however, a whole-patch analysis in pathway-identified striatal SPNs revealed a decrease in miniature EPSCs in heterozygous G2019S KI and R1441C KI mice aged <2 months [116]. Interestingly, the R1441C mutation impacted more dramatically than the G2019S mutation on the glutamatergic synapse since it was associated with greater incorporation of the GluA1 subunits and the AMPA receptors in the membranes of direct pathway SPNs, and larger excitation and protein-kinase A (PKA) activity in this neuron subpopulation [116].

An elevation of GLU transmission is consistent with the recently reported reduction of the levels and activity of excitatory amino acid transporter type 2 (EAAT2), a GLU transporter mainly expressed in astrocytes and responsible for >90% brain GLU reuptake, in the striatum of 4-month-old G2019S KI mice [117]. The reduced EAAT2 activity (*V*_max_ but not *K*_m_) was shown to be due to G2019S-dependent targeting of EAAT2 to recycling/degradation vesicles and consequent reduced trafficking to the neuronal membrane. Strikingly, this process was reversed by MLi-2, revealing the pathogenic role of LRRK2 kinase activity. Moreover, reduction of EAAT2 levels was confirmed in parkinsonian patients carrying the G2019S mutation [117].

Glutamatergic transmission was also investigated using whole-cell patch clamp recording in midbrain DA neurons of 10- to 12 month-old BAC hG2019S mice [36]. The frequency, but not amplitude, of spontaneous EPSCs was lower in mutant compared with non-transgenic mice and this phenotype was reversed by IN-1 [36]. Levels of GluA1 subunits and vesicular glutamate transporter 1 (VGLUT1) were also found to be lower in the midbrain of BAC hG2019S KI mice [36].

## Do LRRK2 rodents model the prodromal phase of PD?

The prodromal or premotor phase of PD is defined as the period when observable signs or symptoms of neurodegeneration appear [3,118,119]. It can precede the diagnosis of PD, hence the motor phase, by 10–20 years. Typical non-motor symptoms in the prodromal phase are RBD, depression, constipation, olfactory dysfunction, and mild cognitive impairment [3,118]. In addition, subtle motor impairment at upper limbs (e.g., impaired tapping and reduced arm swing) can be unveiled if more sensitive tests for complex motor skills are used or objective measures with wearable devices are taken [120–122]. The prodromal phase is clinically heterogeneous [119] and can be shaped by the presence of LRRK2 mutations, such as G2019S. In fact, non-manifesting G2019S carriers show subtle motor changes and higher UPDRS scores, increased constipation and depression, possibly anxiety, and less cognitive impairment or hyposmia compared with healthy individuals [123,124]. These behavioral changes are accompanied by the reduction of PET/SPECT DAT signal that likely reflects early degeneration or functional demise of nigro-striatal DA neurons [84,125,126]. However, PET/SPECT DAT imaging in non-manifest G2019S carriers and healthy controls has yielded conflicting data. In fact, some studies showed a reduced DAT binding in the striatum of non-manifest G2019S carriers [127–130] whereas the analysis of the large cohort of Parkinson’s Progression Markers Initiative (PPMI) patients [123,131] revealed that non-manifest G2019S carriers do not show difference in DAT tracer binding with respect to healthy controls [131]. Moreover, G2019S patients with manifest PD have higher DAT signal compared with sporadic PD patients [123]. Considering the slower progression and the more benign course of LRRK2 PD, one would hypothesize that G2019S somehow slows the degeneration and/or dysfunction of DA synapse. Prodromal PD is also characterized by imaging changes at central and peripheral non-DA synapses which are thought to correlate with the appearance of non-motor symptoms [84,132]. Imaging of the prodromal phase in non-manifest G2019S carriers also revealed higher cortical 5-HT transporter signal [128] and AChE activity [133]. Finally, PD is also characterized by network changes thought to compensates for nigro-striatal degeneration, such as cortical disinhibition [89,134]. This adaptive response reflects an unbalance between cortical glutamatergic excitatory and GABAergic inhibitory transmission [89,134] and has been proposed to occur already in the prodromal phase [89].

To what extent LRRK2 rodents replicate the behavioral and functional changes of prodromal PD? Moderate evidence of olfactory changes, cognitive dysfunction and sleep disturbances in LRRK2 rodents has been collected, despite studies are limited and heterogeneous. On the other hand, evidence of anxiety behavior in LRRK2 rodents remains controversial whereas the occurrence of gastrointestinal dysfunction, depression and pain seems unlikely based on the current studies. From a functional point of view, impairment of DA release and DAT function has been observed across different LRRK2 models, although whether the reduced response to DAT inhibitors or amphetamine is associated with increased [64] or reduced [37] DAT activity needs to be proven. Regarding changes at non-DA synapses, upregulation of cortico-striatal glutamatergic transmission consistently reported in different LRRK2 models might correlate with cortical disinhibition observed in prodromal PD patients. Dysfunctional serotonergic [60] and cholinergic [72,111] transmission has been observed in G2019S mice although these are difficult to reconcile with the changes reported in G2019S non-manifest carriers and detailed above [128,133].

Finally, considering the role of LRRK2 in the immune response, the lack of microgliosis observed in LRRK2 rodents deserves some consideration. Limited evidence of microglial activation in prodromal PD patients has been collected. Imaging studies in idiopathic RBD patients [135] and LRRK2 non-manifest carriers [136] pointed to microglial activation in SN concurrent with nigro-striatal degeneration, although the limited number of LRRK2 patients prevent from generalizations of the results [136]. In fact, no changes in CSF cytokine levels in non-manifesting G2019S carriers has been reported [137,138]. This would suggest that microgliosis is not a feature of the prodromal phase of LRRK2 PD, which is in line with the absence of microgliosis in LRRK2 rodents. In conclusion, prodromal PD is a complex nosological entity characterized by non-motor and mild motor symptoms associated with a validated imaging marker (PET/SPECT DAT) and accompanied by a range of imaging, serum, CSF, tissue and electrophysiological changes that require marker validation [139]. The prodromal phase is heterogeneous and might be differently shaped by LRRK2 mutations [119]. Current studies indicate that LRRK2 rodents partly replicate the spectrum of changes reported in prodromal PD patients although whether these changes really represent early events in a slow process leading to neuronal loss and synuclein pathology or just the impact of the LRRK2 mutations on rodent brain remains a matter for speculation.

## LRRK2 rodents as a useful tool for testing the dual-hit or multiple-hit hypothesis of PD

Sporadic PD is a multifactorial disease where environmental, genetic and individual factors interact to modulate the risk and the progression of the disease and shape its clinical phenotype. A dual-hit hypothesis of PD was initially proposed by Braak and colleagues to explain the PD pathogenesis [140,141]. In their hypothesis, an unknown neurotropic pathogen could enter the brain through the nose and the gut and from these two portals (or ‘hits’) reach the brain anterogradely along the olfactory pathways or retrogradely along the vagal fibers [140,141]. More in general, however, a dual-hit or multiple-hit hypothesis of a multifactorial disease posits that two or more factors, for instance genetic and environmental, concur to determine the pathology. Since LRRK2 mutants carry a genetic risk for PD, a ‘dual-hit’ approach was implemented by exposing LRRK2 rodents to a parkinsonian toxin or trigger. In some studies, age was introduced as an additional variable (‘multiple-hit approach’). Within this frame, hG2019S and hA53T α-syn transgenic mice were generated to assess whether LRRK2 mutant overexpression could accelerate the pathology induced by α-syn mutant. Indeed, hG2019S enhanced the degeneration of striatal SPNs, deposition of α-syn and microgliosis/astrogliosis induced by hA53T α-syn whereas LRRK2 KO mice were protected [57]. Nonetheless, also overexpression of hWT LRRK2 or a LRRK2 KD mutant had the same effect, questioning the pathogenic role of the LRRK2 kinase [57]. A later study using the same approach, however, failed to observe a facilitating effect of hG2019S on hA53T α-syn-induced pathology in several brain nuclei [58]. In this study, a similar number of nigral neurons was found in 13- to 14-month-old hA53T α-syn mice and double hG2019S/A53T α-syn mice, ruling out a possible synergistic effect of the two hits on nigro-striatal degeneration [58]. Lack of hG2019S facilitation on hA53T α-syn-induced pathology and microgliosis was further confirmed allowing the co-expression of both TGs in the same cells via the Thy1 promoter [49]. Given the inconsistencies produced by the double mutant approach, fusion models of synucleinopathy were implemented in LRRK2 rodents. BAC hG2019S rats developed larger nigro-striatal cell loss and microgliosis compared with non-transgenic controls after unilateral intranigral injection of recombinant adeno-associated virus (rAAV) serotype 2/1 (rAAV2/1) α-syn [14]. In this model, the sensitivity conferred by hG2019S was reversed by LRRK2 kinase inhibitor PF-06447475 pointing to a pathogenic involvement of LRRK2 kinase activity [14]. Using the same approach but a different vector (AAV2/9 carrying A53T α-syn) in G2019S KI mice led to same result [142]. In this case, the facilitating effect of G2019S LRRK2 on nigral DA neurons degeneration was only observed in aged mice and was not associated with sustained microgliosis. To model the pathological process of α-syn fibrillization and spreading, preformed fibrils (PFFs) of recombinant α-syn are used [143,144]. Intranigral injection of mouse PFFs caused twice as much abundance of pSer129 α-syn-positive inclusions in the SNc neurons of BAC hG2019S rats compared with non-transgenic controls although no nigro-striatal degeneration was observed in this study [144]. More recently, bilateral intrastriatal injection of mouse α-syn PFFs resulted in early appearance of α-syn inclusions in nigral DA neurons (from 1 month after injection) and larger nigro-striatal degeneration in BAC hG2019S mice compared with non-transgenic controls [75]. Again, this enhanced pathology was associated with a larger microglial and astrocyte activation.

Evidence for enhanced susceptibility of LRRK2 mutants to parkinsonian stressors was provided also in a classical model of mitochondrial parkinsonism, i.e. that induced by complex I inhibitors 1-methyl-4-phenyl-1,2,3,6-tetrahydropyridine (MPTP) and rotenone. It was originally reported that a typical acute MPTP protocol (4 × 18 mg i.p., every 2 hr) caused larger nigro-striatal degeneration and astrocytosis in 3-month-old CMVE-PDGF hG2019S mice with respect to hWT LRRK2 OE and non-transgenic controls [145]. Likewise, subtoxic MPTP (2 × 2.5 mg/kg s.c., every 24 h) caused marked nigral DA neurons and striatal DA terminals loss and astrocyte activation in 4-month-old PrP hG2019S mice, being significantly less effective in hWT LRRK2 OE mice and ineffective in non-transgenic mice [61]. To confirm the neurotoxic role of enhanced LRRK2 kinase activity, systemic IN-1 abolished the neurodegeneration in hWT mice and attenuated it in hG2019S mice. The overall picture was confirmed in G2019S KI mice. First, application of the mitochondrial complex I inhibitor rotenone to striatal slices caused larger reduction of field potential amplitude, an index of neuronal toxicity, in the SPNs of 3-month-old G2019S KI mice compared with WT mice [94]. Moreover, subacute MPTP (25 mg/kg s.c. once daily for seven days) induced larger nigro-striatal degeneration in G2019S KI mice compared with WT, LRRK2 KO and KD mice [15]. This effect was rescued by prophylactic treatment with PF-6447475 or symptomatic treatment with PF-6447475 and MLi-2. Remarkably, while both inhibitors rescued the toxic effect of G2019S on nigral DA cell number, only MLi-2 was able to counteract the toxic effect of G2019S on striatal DA terminals, likely due to the different degree of LRRK2 kinase inhibition achieved in striatum [15]. Finally, LRRK2 mutants also showed larger neurotoxic responses in a model of neuroinflammation-induced PD. Acute administration of lipopolysaccharides (LPS, 5 mg/kg i.p.) in R1441G KI mice and WT controls reduced the number of nigral cells selectively in R1441G KI mice already at 24 h and up to 7 months after administration, which was associated with a larger increase in microglial activation (only at 7 months) [35].

## Concluding remarks

Collectively, rodents carrying pathogenic LRRK2 mutations have construct validity but do not consistently mimic the clinical phase of PD. This is perhaps not too surprising, considering the slow progression and low penetrance of the G2019S mutation in humans. Among rodents carrying pathogenic LRRK2 mutations, however, two different models, i.e transgenic mice based on the CMVE/PDGF and the TH promoter show age-dependent hypokinesia and nigro-striatal DA degeneration, although without typical LB-like α-syn inclusions. The mechanisms underlying the effectiveness of these neurodegeneration-prone models should be clarified to refine PD modeling and understand how LRRK2 shapes PD. Although most LRRK2 rodents do not have face validity as disease models, they present with behavioral signs, such as sleep disturbances, hyposmia and memory deficits, along with functional changes at DA and non-DA pathways that are reminiscent of prodromal PD. In this respect, LRRK2 rodents greatly differ from classical toxin-induced rodent models, where overt nigro-striatal degeneration and hypokinesia are induced by acute administration of 6-OHDA or acute/subacute/chronic administration of MPTP [146–148]. These models, however, do not reproduce the progressive nature (neurodegeneration emerges within days from toxin administration) and the etiology (typical α-syn inclusions are lacking) of PD. Nonetheless, the 6-OHDA model has high predictive validity since all symptomatic antiparkinsonian drugs proved effective in this model. Moreover, being a human parkinsonian toxicant MPTP replicates in mice the mitochondrial dysfunction and the accompanying neurotoxicity observed in PD patients, offering a good model for screening of putative neuroprotective agents. LRRK2 rodents also differ from the other major genetic models of autosomal dominant PD, that is represented mice overexpressing WT or mutant (usually A53T and A30P) hα-syn. As for LRRK2 transgenics, a variety of promoters (TH, Thy-1, PDGF, PrP, to mention the most popular) have been used to obtain optimal levels and patterns of TG expression [149,150]. A stringent phenotypic comparison of LRRK2 rodents and α-syn mice is out of the scope of this review. Nonetheless, very much like LRRK2 rodents, α-syn mice present with a different degree of α-syn pathology, hypokinesia, motor and non-motor signs depending on the promoter used, the TG expressed and the genetic background of the mice. Nigro-striatal degeneration is not a feature of these models although age-dependent loss of striatal DA levels is consistently reported for the Thy1 (‘Line61’) mouse (that overexpresses hWT α-syn under the mouse Thy1 promoter) or the SYN120 mouse (that overexpresses the 1-120 truncated form of human α-syn under the rat TH promoter), suggesting early dysfunction of nigro-striatal DA neurons [150,151]. In conclusion, most LRRK2 rodents will not develop a PD-like synucleinopathy during their life but present with behavioral signs and functional changes that are reminiscent of prodromal PD, suggesting they can model the early cellular pathogenic events set in motion by the LRRK2 mutation. Carrying a genetic risk factor, LRRK2 rodents challenged by a parkinsonian toxin or stressor are an invaluable tool to test the contribution of risk or protective factors in PD pathogenesis.

## Data Availability

N/A

## Abbreviations

α-syn: α-synuclein
5-HT: 5-hydroxytryptamine
5CSRTT: 5-choice serial reaction time task
AChE: acetylcholinesterase
BAC: bacterial artificial chromosome
CaMKII: calcium–calmodulin-dependent protein kinase II
CLOCK: circadian locomoter output cycles *protein* kaput
CMV: cytomegalovirus
CMVE: cytomegalovirus enhanced
DA: dopamine
DAT: dopamine transporter
DOPAC: 3,4-dihydroxyphenylacetic acid
EAAT2: excitatory amino acid transporter type 2
EEG: electroencephalography
EPM: elevated plus maze
EPSCs: extracellular postsynaptic currents
FSCV: fast scan cyclic voltammetry
FST: forced swimming test
GDNF: glial cell line–derived neurotrophic factor
GFAP: glial fibrillary acidic protein
GLU: glutamate
GWAS: genome-wide association studies
HMW: high molecular weight
HVA: homovanillic acid
i.p.: intraperitoneal
IR: immunoreactivity
KD: kinase-dead
KI: knock-in
KO: knock-out
LBs: Lewy bodies
LPS: lipopolysaccharides
LRRK2: leucine-rich repeated kinase 2
MPTP: 1-methyl-4-phenyl-1,2,3,6-tetrahydropyridine
NA: noradrenaline
NOR: novel object recognition
OE: overexpressors
OF: open field
PD: Parkinson’s disease
PDGF: platelet-derived growth factor
PET: positron emission tomography
PFCx: prefrontal cortex
PFFs: pre-formed fibrils
Pitx3: paired-like homeodomain transcription factor 3/pituitary homeobox 3
PKA: protein-kinase A
PLC: phospholipase C
PrP: prion protein
pSer129: phosphorylated Serine 129
Ptau: phosphorylated tau
rAAV: recombinant adeno-associated virus
REM: rapid eye movement
s.c.: subcutaneous
Shh: sonic hedgehog
SNc: substantia nigra compacta
SPNs: striatal projection neurons
TetO: tetracycline operator
TG: transgene
TH: tyrosine hydroxylase
Thy1: thymus cell antigen 1
TSPO: translocator protein
TST: tail suspension test
UB: ubiquitin
VGLUT1: vesicular glutamate transporter 1
VMAT2: vesicular monoamine transporter 2
WT: wild-type
